# Self-reported binary gender prediction from personality traits: Alignment between machine learning importance and classical effect sizes

**DOI:** 10.1371/journal.pone.0355193

**Published:** 2026-08-07

**Authors:** Heeseung Cho, Yiyu Chen, Christian Wallraven

**Affiliations:** 1 Department of Artificial Intelligence, Korea University, Seoul, Republic of Korea; 2 Department of Brain and Cognitive Engineering, Korea University, Seoul, Republic of Korea; The University of Aukland, NEW ZEALAND

## Abstract

Gender differences in personality have been characterized using effect sizes such as Cohen’s *d* and multivariate measures such as the Mahalanobis distance. However, as machine learning models are increasingly applied to personality classification, it remains unclear how model-based feature importance relates to these classical measures of group separation. This study examined this relationship using two large-scale personality datasets comprising 18,062 participants in the Big5 and 44,324 participants in the 16PF. We trained multiple linear and nonlinear machine learning models (Logistic Regression, Random Forest, Extra Trees, LightGBM, and Explainable Boosting Machine) to predict self-reported binary gender from trait scores and derived feature-importance rankings using SHAP and permutation importance. These rankings were compared with univariate effect sizes, standardized linear discriminant weights, and trait-level contributions to the Mahalanobis distance. Across 20 model–resampling configurations, trait-importance rankings were highly stable in both datasets. In the Big5, univariate effect size rankings closely matched model-based importance rankings, with rank correlations of 0.98 to 1.00. Conversely, in the 16PF, alignment between univariate effect size and model-based importance was moderate (ρ=0.56−0.64), whereas alignment with multivariate discriminant structure remained strong (ρ=0.83−0.85). These findings indicate that divergence between univariate and model-based importance is consistent with covariance-mediated multivariate weighting rather than the emergence of novel predictive structure. When trait intercorrelations are limited, univariate summaries approximate multivariate importance; when covariance is substantial, multivariate geometry reshapes trait contributions, and model-based explainability metrics closely track the resulting discriminant structure.

## Introduction

Gender differences in personality traits have long been a central topic in personality psychology. Although broad consensus exists that men and women differ on certain personality dimensions, the magnitude, structure, and interpretation of these differences remain debated [1,2]. Competing accounts attribute observed differences to sociocultural influences, biological factors, or their interaction [3,4]. Clarifying how gender differences are quantified and interpreted therefore remains of fundamental importance.

Traditionally, gender differences in personality have been summarized using univariate effect sizes, most commonly Cohen’s *d* [5] – some meta-analytic reviews have emphasized that many observed differences are small in magnitude [1,6]. Within standardized personality frameworks such as the Five-Factor Model (Big5) [7], consistent differences have been reported for traits such as Neuroticism and Agreeableness [4,8,9]. In the Sixteen Personality Factor Model (16PF), somewhat larger differences have been observed for traits including Sensitivity, Anxiety, and Warmth [10]. However, univariate effect sizes quantify marginal mean differences and do not account for correlations among traits.

To address this limitation, Del Giudice and colleagues proposed the Mahalanobis distance (*D*) as a multivariate measure of gender differentiation that incorporates covariance structure [11,12]. Under this framework, relatively modest univariate effects have been shown to correspond to substantially larger multivariate separation [10,12–14]. Subsequent methodological work has emphasized that multivariate effect sizes capture not only mean differences but also covariance structure among traits, and that trait intercorrelations can substantially reshape interpretations of group differentiation [15,16]. These findings suggest that the interpretation of gender differences in some cases depends critically on multivariate trait structure rather than on marginal effects alone.

In parallel, machine learning (ML) methods have become increasingly prominent in personality research [17]. Empirical studies have demonstrated that personality traits can be inferred from digital footprints and behavioral traces with substantial accuracy [18–20]. Those settings are the targeted application domain of importance-based interpretability; the present work operates one step earlier, validating under a controlled, additively factorised feature design that feature importance does in fact recover established statistical effects before such methods are transported to sparse, behavioral-trace regimes. As predictive models become more widely adopted, questions of interpretability have gained importance alongside classification accuracy [21]. Beyond overall classification performance, recent advances in explainable ML provide tools for estimating feature importance within multivariate predictive models. For example, permutation importance [22] quantifies the decrease in classification performance when a feature is disrupted, whereas SHapley Additive exPlanations (SHAP) [23,24] provide a game-theoretic decomposition of model predictions into additive feature contributions. These methods are often used to estimate how strongly individual traits contribute to predictions within correlated predictor spaces [24].

Conceptually, both classical multivariate statistics and ML-based explainability can be interpreted as quantifying trait contributions within correlated predictor spaces. Linear discriminant analysis (LDA), for example, derived from classical statistical geometry [25], characterizes the direction of maximal group separation under covariance structure. Model-based explainability methods, in contrast, arise from algorithmic attribution frameworks that decompose predictive functions into feature-level contributions [23]. Despite these conceptual parallels, their direct empirical correspondence has received limited systematic investigation. It remains unclear whether ML-based importance measures reproduce classical multivariate weighting or capture patterns that diverge from known statistical geometry.

The present study addresses this question directly: do ML-based feature-importance rankings recover classical multivariate separation structure, or do they capture patterns that diverge from known statistical geometry? We examine this using two large-scale personality datasets based on the Big5 and the 16PF, which differ in dimensionality and inter-trait correlation structure. We train multiple ML models to predict gender from personality traits and derive feature-importance rankings using SHAP and permutation importance. These rankings are compared with univariate effect sizes, multivariate discriminant weights, and trait-level contribution terms derived from Mahalanobis distance decomposition. By examining both the stability of importance rankings across models and their structural correspondence with classical measures, this study evaluates whether model-based representations recover population-level multivariate geometry. In doing so, we clarify the conditions under which univariate summaries approximate multivariate importance and when covariance structure reshapes trait contributions.

## Materials and methods

### Datasets

We used publicly available datasets obtained from the open-source psychometrics project (OpenPsychometrics; https://openpsychometrics.org/), which hosts large-scale personality questionnaire data collected from voluntary, anonymized participants. Data were accessed for research purposes beginning on 1 December 2025. All accessed data were anonymized survey responses, and no information that could identify individual participants was available during or after data collection.

From this resource, we selected two widely used personality instruments that provide both personality measures and a self-reported binary gender label (Male / Female): Big5 and the 16PF. We distinguish this variable from biological sex and from contemporary multi-category gender constructs. These two instruments differ substantially in dimensionality and inter-trait correlation structure, allowing examination of how trait structure influences the correspondence between marginal effect sizes and multivariate importance.

#### Big5.

The Big5 model of personality was originally formalized by Goldberg [7] and is one of the most widely accepted frameworks in personality psychology. It comprises five broad dimensions: Extraversion, Neuroticism, Agreeableness, Conscientiousness, and Openness to Experience.

The Big5 dataset was based on the International Personality Item Pool (IPIP) FFM-50 questionnaire, consisting of 50 Likert-type items rated on a five-point scale. Previous studies have demonstrated measurement equivalence of the IPIP Big5 across gender groups [26]. The original dataset included responses from 19,719 participants and was last updated in May 2014.

#### 16PF.

The 16PF was developed by Cattell to capture finer-grained dimensions of personality derived through factor-analytic methods [27]. The 16PF questionnaire assesses 16 primary personality traits using a total of 163 items.

The 16PF dataset, last updated in May 2014, included responses from 49,159 participants prior to preprocessing. Consistent with prior large-scale analyses using the same data source [10], one primary factor was excluded due to measurement limitations in the online questionnaire, resulting in a final set of 15 personality traits used for analysis.

#### Data cleaning.

We followed established large-scale analyses of gender differences in personality [10,12] to ensure data quality and methodological consistency across datasets. Respondents were excluded if they met any of the following criteria: (1) 25 or more missing responses, (2) uniform responding across all items (e.g., selecting only the minimum or maximum scale value), or (3) self-reported age outside the range of 16–90 years. Because the study focused on binary gender classification, only participants who self-identified as male or female were retained; records with other or unspecified gender categories were excluded due to limited representation. For the 16PF dataset, an additional response accuracy index was available. Participants with invalid accuracy scores were excluded, and only those with an accuracy score of at least 50% were retained, consistent with prior recommendations [10].

After cleaning, the final sample comprised 44,324 participants in the 16PF (59.6% female) and 18,062 participants in the Big5 (60.7% female). Summary statistics are reported in Table 1.

**Table 1:** Descriptive statistics and gender effect sizes for the Big5 and 16PF datasets. Score values are reported as mean ± standard deviation. |*D*|: absolute Mahalanobis distance, |*d*|: absolute Cohen’s *d*, α: Cronbach’s alpha. Bold |*d*| values indicate the largest effect size within each dataset.

Dataset	Feature	Raw score	|*d*|	α
Big5 N = 18,062 |*D*| = 0.684	E. Extraversion	3.028±0.919	0.126	0.893
	A. Agreeableness	3.866±0.705	**0.468**	0.831
	C. Conscientiousness	3.370±0.727	0.078	0.812
	N. Neuroticism	3.078±0.862	0.321	0.872
	O. Openness	3.913±0.621	0.219	0.795
16PF N = 44,324 |*D*| = 1.108	A. Warmth	3.708±0.620	0.304	0.822
	C. Emotional Stability	3.355±0.752	0.315	0.852
	E. Assertiveness	3.515±0.626	0.214	0.814
	F. Gregariousness	3.308±0.664	0.002	0.783
	G. Dutifulness	3.158±0.715	0.249	0.810
	H. Friendliness	3.202±0.848	0.025	0.897
	I. Sensitivity	3.505±0.582	**0.786**	0.662
	L. Distrust	2.851±0.690	0.024	0.847
	M. Imagination	3.469±0.629	0.037	0.772
	N. Reserve	2.934±0.812	0.110	0.871
	O. Anxiety	3.152±0.749	0.482	0.844
	Q1. Complexity	3.794±0.577	0.157	0.758
	Q2. Introversion	3.343±0.671	0.034	0.831
	Q3. Orderliness	3.170±0.650	0.037	0.778
	Q4. Emotionality	2.812±0.667	0.096	0.798

### Machine learning training framework

ML models were used to evaluate whether model-based importance corresponds to the multivariate structure underlying gender separation. Data were split into stratified training (90%) and test (10%) sets by gender.

We evaluated Logistic Regression (LR) as a linear baseline, and Random Forests (RF) [28], Extremely Randomized Trees (ET) [29], LightGBM (LGB) [30], and the Explainable Boosting Machine (EBM) [31] as non-linear ensemble models. This set spans linear, tree-based, and additive frameworks, allowing assessment of whether importance alignment generalizes across classifier families.

Hyperparameter optimization for LR, RF, ET, and LGB was performed exclusively within the training data using randomized search over 50 candidate configurations, each evaluated by 10-fold stratified cross-validation. Within each fold, resampling and z-score normalization were fitted on the training subset only, followed by model fitting; validation data were transformed using normalization parameters estimated from the corresponding training fold and were never resampled. EBM was fit without hyperparameter tuning, using the same preprocessing pipeline with interactions fixed to zero.

After model selection, each final pipeline was refit on the full training set and evaluated on the held-out test set, where normalization was applied without resampling.

To assess robustness to class imbalance, four resampling conditions were evaluated: no resampling, Synthetic Minority Over-sampling Technique (SMOTE) [32], TomekLinks under-sampling [33], and SMOTE–TomekLinks. Resampling was confined to training data throughout. Hyperparameter search spaces, cross-validation settings, and detailed reproducibility information, including software versions and random seed specification, are provided in S1 Text. Full implementation code are provided in S1 File.

### Evaluation metrics and explainability

Given the moderate class imbalance present in both datasets, classification performance was primarily evaluated using the area under the receiver operating characteristic curve (AUC), which is robust to class imbalance [34]. Accuracy and F1-score were reported as secondary metrics.

To interpret model predictions, we employed two complementary, model-agnostic feature importance methods. Permutation importance quantifies the decrease in classification performance when a feature’s values are randomly permuted, capturing its contribution to classification accuracy [22]. When features are correlated, permutation importance may underestimate individual contributions because correlated features partially preserve the permuted feature’s information [35,36]; this property is relevant to the 16PF, where inter-trait correlations are substantial. SHAP values provide a game-theoretic decomposition of predictions into additive feature contributions, enabling both global importance estimation and directional interpretation [23]. SHAP values were computed using model-appropriate explainers: TreeExplainer for tree-based models (RF, ET, LGB), LinearExplainer for LR, and the model-agnostic permutation-based Explainer for EBM.

All feature importance analyses were conducted exclusively on held-out test data to avoid optimistic bias and data leakage. In this study, Cohen’s *d* was interpreted as a measure of marginal group differences, whereas feature importance estimates were interpreted as conditional contributions within a multivariate framework.

### Statistical analysis

#### Population-level separation structure.

Marginal gender differences for each trait *i* were quantified using Cohen’s $d_{i}$, defined as the standardized mean difference between female and male participants:


$$\begin{array}{c} d_{i}=\frac{M_{f,i}-M_{m,i}}{s_{\text{pooled},i}}. \end{array}$$ (1)

Let $d=(d_{1},...,d_{p}{)}^{⊤}$ denote the vector of standardized mean differences across the *p* traits. Absolute values $\left|d_{i}\right|$ were used to rank traits by marginal effect magnitude [5,37].

To summarize global gender differences while accounting for covariance among traits, we computed the Mahalanobis distance (*D*) [11,12,15]:


$$\begin{array}{c} D=\sqrt{\left(\Delta \mu {)}^{⊤}\Sigma_{\text{pooled}}^{-1}(\Delta \mu \right)}, \end{array}$$ (2)

where $\Delta \mu =\mu_{f}-\mu_{m}$ and $\Sigma_{\text{pooled}}$ is the pooled within-group covariance matrix. *D* was computed separately for the Big5 and 16PF datasets using all traits in each instrument. Pooled within-group correlation matrices for both datasets are reported in S1 Table.

While $\left|d_{i}\right|$ and *D* quantify marginal and global separation magnitude, respectively, they do not describe the directional structure of multivariate separation. To characterize this structure, linear discriminant analysis (LDA) was applied [25], yielding the discriminant vector


$$\begin{array}{c} w=\Sigma_{\text{pooled}}^{-1}\Delta \mu . \end{array}$$ (3)

In this study, LDA was used solely to obtain interpretable discriminant weights and not for classification performance evaluation. Classification itself relies on LR—whose asymptotic justification requires only that the conditional log-odds be linear in the features, not multivariate normality—together with four nonlinear, distribution-free classifiers. Formal multivariate-normality and equal-covariance diagnostics (Box’s M, Mardia, Henze–Zirkler) are therefore reported in S1 Table as a transparency check rather than as validation of the inference engine.

To connect discriminant direction with trait-level contributions, we adopted a standardized parameterization. Let 𝐒 be the diagonal matrix of pooled within-group standard deviations and ${𝐑}_{\text{pooled}}={𝐒}^{-1}\Sigma_{\text{pooled}}{𝐒}^{-1}$ the pooled within-group correlation matrix. The standardized discriminant weights were defined as


$$\begin{array}{c} \tilde{w}=𝐒w={𝐑}_{\text{pooled}}^{-1}d. \end{array}$$ (4)

Under this formulation, the squared Mahalanobis distance decomposes as [15,38]


$$\begin{array}{c} D^{2}=d^{⊤}\tilde{w}=\sum_{i=1}^{p}d_{i}{\tilde{w}}_{i}. \end{array}$$ (5)

Trait-level contributions were defined as $c_{i}=d_{i}{\tilde{w}}_{i}$, and absolute values $\left|c_{i}\right|$ were used to rank traits by their contribution to global multivariate separation.

These classical measures provide specific theoretical expectations for comparison with model-based importance. For a linear classifier operating on standardized features, SHAP values reduce to standardized coefficient magnitudes [23], which in binary classification are proportional to LDA discriminant weights. This motivates comparing SHAP importance rankings with $\left|{\tilde{w}}_{i}\right|$. Separately, permutation importance quantifies the decrease in classification performance when a feature is disrupted, which reflects that feature’s contribution to overall separability. If total multivariate separation decomposes as $D^{2}=\sum_{i}c_{i}$, then permuting trait *i* disrupts its contribution $c_{i}$ to global separation magnitude; under this reasoning, permutation importance would be expected to track $\left|c_{i}\right|$ rather than $\left|d_{i}\right|$ or $\left|{\tilde{w}}_{i}\right|$ alone. These theoretical expectations—SHAP with discriminant weighting ($\left|\tilde{w}\right|$) and permutation importance with separation contribution ($\left|c_{i}\right|$)—motivated the specific alignment comparisons tested below.

#### Model evaluation and rank-based analyses.

Classification performance was evaluated across ML models and resampling strategies using accuracy, AUC, and F1-score. To assess whether performance differed systematically across models independent of resampling strategy, Friedman tests were conducted with model as the main factor, pooling results across resampling conditions [39,40]. These tests determined whether differences justified selection of a representative model for subsequent analyses.

To evaluate the stability of feature-importance rankings across modeling configurations, Kendall’s coefficient of concordance (*W*) was computed [41]. Traits were treated as ranked items and each model–resampling combination as an independent rater.

All alignment analyses were conducted on absolute values and compared via ranks rather than raw magnitudes. Absolute values were used because the measures compared here differ in sign conventions: Cohen’s *d* and LDA weights depend on group coding order, SHAP values depend on the reference class, and permutation importance is inherently non-negative. Taking absolute values reduces all measures to a common question—how strongly each trait contributes—independent of directional coding. Rank-based comparison was adopted because the measures also differ in scale and units, making direct magnitude comparison uninformative. Alignment between classical and ML-based measures was therefore assessed using Spearman’s rank correlation (ρ). For each configuration, SHAP importance was computed as the mean absolute SHAP value per trait on the held-out test set, and permutation importance as the decrease in test-set AUC after permuting each trait. These values, together with $\left|d_{i}\right|$, $\left|{\tilde{w}}_{i}\right|$, and $\left|c_{i}\right|$, were converted to trait-level ranks;the model-based ranks were then aggregated across configurations into a single consensus ranking, and Spearman’s ρ was computed between this aggregated ranking and each classical ranking. Mean aggregation was used in the primary analysis, with median aggregation reported as a robustness check.

#### Multicollinearity diagnostics.

Because SHAP and single-feature permutation importance are sensitive to correlated predictors [42], we quantified inter-trait dependence for each trait feature set (max/median |*r*|, condition number and Belsley condition index, eigenvalue-entropy effective dimensionality, and variance inflation factor; S1 Table). As robustness checks we additionally computed conditional (group) permutation importance [35,43,44] and cluster-level (grouped) SHAP [45]: traits were hierarchically clustered by Ward linkage on the Spearman correlation distance $d=1-|r_{s}|$, the dendrogram was cut at *d* = 0.7, each cluster was permuted jointly to preserve within-cluster correlation, and mean(|SHAP|) was summed within clusters. Cluster-level importance rankings were compared across models with Spearman’s ρ (S1 Table).

## Results

The following lists the results of our analyses.

### Classification performance

Classification performance was evaluated using test-set AUC (Table 2). Across model classes, mean AUC was 0.789–0.798 in the 16PF (mean: 0.793) and 0.678–0.684 in the Big5 (mean: 0.680), yielding a dataset-level difference of 0.113. This ordering parallels the larger global multivariate gender separation observed in the 16PF (*D* = 1.108) relative to the Big5 (*D* = 0.684).

**Table 2:** Classification performance across ML models for the Big5 and 16PF datasets. Values are reported as mean and ± standard deviation across four resampling conditions (no resampling, SMOTE, TomekLinks, and SMOTE–TomekLinks). Standard deviations below 0.0005 are reported as 0.000 due to rounding.

Dataset	Model	AUC	Accuracy	F1
Big5	LR	0.678 ± 0.000	0.643 ± 0.017	0.614 ± 0.014
	RF	0.679 ± 0.003	0.647 ± 0.014	0.618 ± 0.011
	ET	0.684 ± 0.001	0.643 ± 0.011	0.613 ± 0.021
	LGB	0.681 ± 0.002	0.646 ± 0.008	0.619 ± 0.012
	EBM	0.678 ± 0.004	0.654 ± 0.013	0.614 ± 0.004
16PF	LR	0.790 ± 0.000	0.719 ± 0.006	0.709 ± 0.001
	RF	0.794 ± 0.001	0.726 ± 0.002	0.715 ± 0.001
	ET	0.795 ± 0.002	0.727 ± 0.001	0.714 ± 0.005
	LGB	0.798 ± 0.002	0.730 ± 0.002	0.719 ± 0.002
	EBM	0.789 ± 0.000	0.727 ± 0.001	0.712 ± 0.002

Although Friedman tests detected statistically significant model effects (Big5: $\chi^{2}(4)=10.60$, *p* = 0.031; 16PF: $\chi^{2}(4)=14.00$, *p* = 0.007), we stress that the magnitude of these differences was *small*. In the 16PF, the AUC range across models was 0.009 (0.798–0.789), and in the Big5 it was 0.006 (0.684–0.678), indicating highly limited practical variation based on model choice.

Choice of resampling method to correct for class imbalance had a negligible effect on model accuracies in the 16PF($\chi^{2}(3)=0.60$, *p* = 0.896). Although a nominal resampling effect was detected in the Big5 ($\chi^{2}(3)=8.28$, *p* = 0.041), the associated AUC range was small (0.004). Overall, differences across models and resampling strategies were modest relative to the dataset-level separation, supporting subsequent analyses based on aggregated importance rankings. Full performance metrics for all 20 model–resampling configurations are reported in S1 Table.

### Importance stability across configurations

To determine whether trait-importance rankings were sensitive to modeling choices, cross-configuration concordance was quantified using Kendall’s *W* (Table 3). Trait rankings exhibited strong agreement across the 20 model–resampling configurations for both SHAP and permutation importance. Concordance was high in the Big5 (*W* = 0.950 for SHAP; *W* = 0.991 for permutation) and remained substantial in the 16PF (*W* = 0.845 for SHAP; *W* = 0.878 for permutation), with all tests reaching statistical significance (*p* < 0.001).

**Table 3:** Cross-configuration concordance of trait-importance rankings across 20 model–resampling combinations.

Dataset	Method	Kendall’s *W*	df	*p*
Big5	SHAP	0.950	4	< .001
Big5	Permutation	0.991	4	< .001
16PF	SHAP	0.845	14	< .001
16PF	Permutation	0.878	14	< .001

These findings indicate that importance rankings are largely invariant across classifier families and resampling strategies, justifying the use of configuration-averaged rankings in alignment analyses. Complete trait-level importance rankings for each configuration are provided in S1 Table.

Inter-trait correlations were modest in both feature sets (maximum |*r*| = 0.34 in the Big5 and 0.75 in the 16PF; maximum variance inflation factor 1.22 and 3.35; Belsley condition index 1.72 and 4.58, both below the standard threshold of 10; S1 Table). Conditional (group) permutation importance and cluster-level grouped SHAP confirmed that, although SHAP and single-feature permutation redistribute credit within correlated clusters, the cluster-level importance ordering remained fully consistent across the five classifiers (S1 Table). The reported trait-importance rankings are therefore not artifacts of collinearity.

### Alignment with classical separation measures

Having established that both classification performance and trait-importance rankings are stable across configurations, we examined how model-based importance aligns with classical measures of group separation. For each configuration, SHAP and permutation importance scores were converted to ranks and correlated with absolute univariate effect size (|*d*|), multivariate discriminant weight ($\left|\tilde{w}\right|$), and the joint multivariate contribution term ($\left|c_{i}|=|d_{i}{\tilde{w}}_{i}\right|$). For each importance measure, trait rankings were aggregated across the 20 configurations and a single Spearman correlation was computed against each classical measure (Table 4); the median-aggregated and per-configuration variants are consistent, which are reported in S1 Table.

**Table 4:** Spearman rank correlations between configuration-aggregated importance rankings and classical separation measures. Each importance measure was summarized by its mean rank across the 20 model–resampling configurations, and a single Spearman correlation was computed against each classical measure. Significance levels: ${\;}^{*}p<\mathrm{.05}$, ${\;}^{**}p<\mathrm{.01}$, ${\;}^{***}p<\mathrm{.001}$.

Comparison	Big5 (*N* = 5)	16PF (*N* = 15)
SHAP vs |*d*|	0.975*	0.564*
SHAP vs $\left|\tilde{w}\right|$	0.975*	0.846***
Perm vs |*d*|	1.000*	0.643**
Perm vs $\left|c_{i}\right|$	1.000*	0.826***

**Fig 1:**
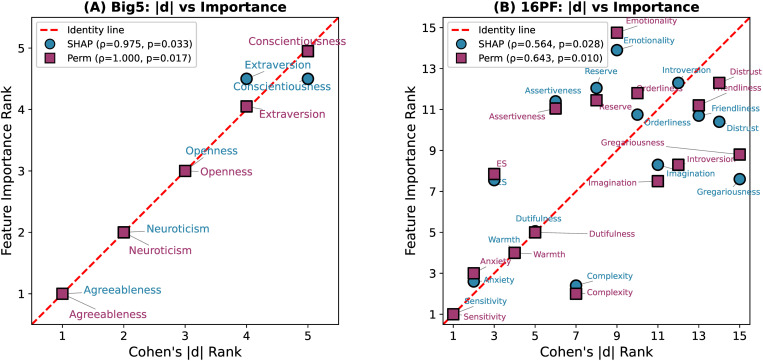
Rank correspondence between univariate effect size (|*d*|) and model-based importance in the Big5 and 16PF. Scatter plots show Spearman rank correlations between absolute Cohen’s *d* and aggregated feature-importance rankings derived from SHAP and permutation importance for (A) Big5 and (B) 16PF. The dashed line denotes the identity line (perfect rank agreement).

In the Big5, model-based importance closely mirrored univariate separation: SHAP and permutation rankings correlated with |*d*| at ρ=0.98 and ρ=1.00, respectively (Fig 1A). Alignment with multivariate discriminant weights was similarly strong (ρ=0.98), indicating minimal divergence between marginal and multivariate rankings.

**Fig 2:**
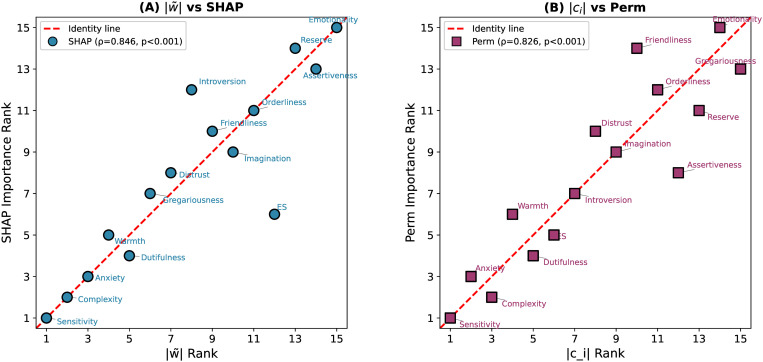
Alignment between multivariate discriminant structure and model-based importance in the 16PF. (A) Rank-level correspondence between standardized discriminant weights ($\left|\tilde{w}\right|$) derived from linear discriminant analysis and SHAP importance. (B) Rank-level correspondence between trait-level contribution terms ($\left|c_{i}|=|d_{i}{\tilde{w}}_{i}\right|$) from the Mahalanobis *D*^2^ decomposition and permutation importance. Each point represents one trait. The dashed red line indicates the identity line (perfect rank agreement). Spearman rank correlations demonstrate strong alignment between multivariate structure and model-based importance.

In contrast, the 16PF exhibited weaker correspondence between |*d*| and importance rankings (ρ=0.56 for SHAP; ρ=0.64 for permutation; Fig 1B). However, alignment with multivariate structure remained strong: SHAP correlated with $\left|\tilde{w}\right|$ at ρ=0.85, and permutation importance with $\left|c_{i}\right|$ at ρ=0.83 (Fig 2). These results indicate that, in the 16PF, importance rankings correspond more closely to multivariate discriminant structure than to marginal effect size.

Because rank correlations discard magnitude, we repeated the alignment analysis on L1-normalized continuous values: within each (model × resampling) cell we L1-normalized the per-trait |*d*|, $\left|\tilde{w}\right|$, mean(|SHAP|), and permutation importance and computed Pearson *r* and top-5 Jaccard overlap (Table 5; per-cell values in S1 Table). The continuous-value correlations are uniformly at least as high as the rank-based values of Table 4, confirming the rank conversion was conservative rather than optimistic. Critically, replacing the univariate |*d*| with the multivariate discriminant weight $\left|\tilde{w}\right|$ restores near-perfect correspondence for the 16PF (median Pearson *r* = 0.987 for SHAP, 0.940 for permutation), indicating that the |*d*|–importance gap in the 16PF reflects the inter-trait correlation structure the model uses correctly, not a misreading of the data.

**Table 5:** Continuous-value correspondence between classical separation measures and model-based importance. Per (model × resampling) cell, |*d*|, $\left|\tilde{w}\right|$, mean(|SHAP|), and permutation importance were L1-normalized; Pearson *r* and top-5 Jaccard overlap are reported as the median across the 20 cells per dataset.

Dataset	Effect	*r*(SHAP)	*r*(Perm)	J_5_(SHAP)	J_5_(Perm)
Big5 / trait	|*d*|	0.989	0.987	1.00	1.00
Big5 / trait	$\left|\tilde{w}\right|$	0.983	0.960	1.00	1.00
16PF / trait	|*d*|	0.811	0.795	0.67	0.67
16PF / trait	$\left|\tilde{w}\right|$	0.987	0.940	1.00	1.00

At the trait level, this divergence is illustrated by the personality trait of *Complexity*, which occupies a mid-range position under |*d*| yet ranks among the highest under both SHAP and permutation importance, consistent with its elevated $\left|\tilde{w}\right|$ and $\left|c_{i}\right|$. Several additional traits with relatively small |*d*| similarly exhibit non-negligible permutation importance. In contrast, comparable rank reordering is minimal in the Big5, where univariate and multivariate rankings already closely align (Fig 1A).

## Discussion

The present study evaluated whether model-based feature importance recovers population-level multivariate structure underlying gender differences in personality traits. The results indicate that it does: across two datasets differing in dimensionality and covariance structure, model-based importance rankings aligned with classical multivariate discriminant geometry rather than with univariate effect sizes alone. This alignment was robust across multiple classifier families and resampling strategies.

The correspondence we report between feature importance and linear statistical metrics is partly a consequence of our data regime. Both datasets satisfy a large observations-per-feature ratio at the trait level used here — *N*/*p* = 3,612 (Big5) and 2,955 (16PF) — more than three orders of magnitude above unity, well into the data-rich regime in which the inductive bias of the learning algorithm carries less weight [46]. Moreover, the Big5 and 16PF are by design additive linear factorizations of personality variance, so the discriminant boundary in this basis is well-approximated by a hyperplane; this is confirmed by the near-identical test AUCs of LR and the tree-/boosting-based models (0.678 for LR vs. 0.679 for RF in the Big5 trait set, and 0.790 vs. 0.798 for the 16PF trait set; Table 2). Our finding that machine-learning feature importance is highly correlated with Cohen’s *d*, the standardized discriminant weight $\tilde{w}$, and Mahalanobis distance is therefore partly anticipated by the dense, linear nature of the problem; it does not entitle a generalization to sparse feature regimes ($\begin{array}{c} p≳N \end{array}$) or to nonlinear decision-boundary problems such as those arising from digital footprints or behavioral traces. The mechanisms we describe — credit redistribution among correlated traits, suppressor effects, multivariate vs. univariate divergence — are likely to translate to those settings, but the specific quantitative correspondence between importance and effect size is not.

The convergence observed here is notable because LDA weights, SHAP values, and permutation importance arise from distinct computational frameworks. LDA derives from classical discriminant analysis under covariance assumptions [25], SHAP from cooperative game-theoretic attribution [23,24], and permutation importance from perturbation-based performance evaluation [22]. Despite these differences, trait rankings converged toward the same multivariate structure. This cross-paradigm correspondence supports the conclusion that classical discriminant geometry remains recoverable under heterogeneous modeling approaches. We note that this correspondence is demonstrated at the level of trait rankings; it does not imply numerical equivalence between importance magnitudes and discriminant weights, but rather that the *ordinal* structure of multivariate separation is preserved across methods.

In the Big5, univariate and multivariate importance rankings closely converged. This may be expected given the modest inter-trait correlations in this dataset (maximum |*r*| = 0.34): when the pooled within-group correlation matrix approximates the identity, ${𝐑}_{\text{pooled}}^{-1}$ introduces negligible reweighting and standardized discriminant weights reduce toward univariate effect sizes ($\tilde{w}\propto d$). Since model-based importance tracks these discriminant quantities (as established above), all three ranking systems—univariate, multivariate, and model-based—converge. This pattern is consistent with cross-cultural evidence of stable gender differences in traits such as Neuroticism and Agreeableness [4,6,8,9].

In contrast, the 16PF revealed systematic divergence between univariate and multivariate rankings. The rank reordering observed for traits such as *Complexity*—where moderate univariate separation coexisted with high multivariate importance—is characteristic of suppressor-like configurations, in which intercorrelations among predictors alter apparent variable importance [47,48]. More generally, methodological work on Mahalanobis-based effect sizes has emphasized that covariance structure can substantially reshape interpretations of group differentiation [15,16]. These results suggest that model-based importance captures this covariance-driven geometry, tracking known discriminant structure [13,14] rather than introducing novel predictive patterns.

The contrast between the Big5 and the 16PF clarifies a structural boundary condition. Because the discriminant weight vector is $\tilde{w}={𝐑}_{\text{pooled}}^{-1}d$, the degree to which multivariate weighting departs from univariate ordering depends on the off-diagonal structure of the correlation matrix [15]. When these off-diagonal entries are negligible—as in the Big5—the ordering of $\left|\tilde{w}\right|$ mirrors |*d*|; when they are substantial, the inverse correlation matrix reweights traits strongly, producing suppressor-like rank reordering. The decisive factor is therefore not dimensionality, nor even the overall strength of the correlations, but their topology — which traits covary, and with what sign — since this configuration is what ${𝐑}_{pooled}^{-1}$ propagates into the up- and down-weighting of traits and the emergence of suppressor effects.

A further implication concerns the distinction between explainability metrics. SHAP importance aligned most closely with standardized discriminant weights ($\left|\tilde{w}\right|$), capturing discriminant weighting structure. As outlined in the Methods, this correspondence has a direct theoretical basis: under a linear model with standardized inputs, SHAP values reduce to standardized coefficients proportional to LDA weights [23,25]. The empirical result indicates that this relationship extends, in attenuated form, to the non-linear classifiers examined here only because they are applied to data with a near-linear discriminant boundary, as in the present additively linear feature spaces; it does not establish that the correspondence holds for genuinely nonlinear decision-boundary problems (see below). Permutation importance, in contrast, tracked joint contribution terms ($\left|c_{i}\right|$), reflecting contribution to overall separation magnitude (*D*^2^) under the Mahalanobis decomposition framework [15]. This alignment is expected because permuting a trait removes its contribution to total separability; because $D^{2}=\sum_{i}c_{i}$, the resulting performance loss should be proportional to $\left|c_{i}\right|$ rather than to the marginal effect $\left|d_{i}\right|$ or the discriminant weight $\left|{\tilde{w}}_{i}\right|$ alone. These results indicate that SHAP and permutation importance quantify complementary aspects of multivariate geometry: the former tracks discriminant weighting ($\left|\tilde{w}\right|$), and the latter tracks each trait’s contribution to overall group separation ($\left|c_{i}\right|$). The appropriate metric therefore depends on whether the inferential focus concerns discriminant weighting or total separation contribution.

Given that the SHAP–$\left|\tilde{w}\right|$ correspondence is mathematically exact under a linear model, we examined whether excluding the linear model (LR) altered the alignment patterns (S1 Table). In the Big5, results were unchanged. In the 16PF, SHAP alignment with $\left|\tilde{w}\right|$ decreased from ρ=0.846 to ρ=0.756—consistent with the removal of a mathematically constrained model—but remained substantial. Permutation importance alignment was unchanged (ρ=0.828), and SHAP alignment with |*d*| improved (ρ=0.564 to 0.681). These results indicate that non-linear models independently recover the majority of the discriminant structure, and that the convergence reported in the main analysis is not solely attributable to the linear model. This convergence is consistent with the observation that all models—linear and non-linear—operate on the same population-level covariance structure; when that structure strongly shapes the classification boundary, even flexible learners approximate the LDA-optimal direction. This does not imply the absence of non-linear interaction effects; rather, it indicates that trait-level marginal importance is dominated by the additive discriminant structure even when models are capable of capturing higher-order patterns.

These findings have practical implications for personality research. Univariate effect sizes remain informative when trait intercorrelations are limited [5]. However, in more highly structured trait systems, multivariate or model-based importance measures provide a more complete account of how traits jointly contribute within correlated configurations. Rather than positioning ML as a replacement for classical statistics, the present results support a complementary interpretation: explainability metrics can serve as alternative operationalizations of classical multivariate separation.

### Limitations

Several constraints qualify the scope of our findings.

***Inter-trait correlations and importance sensitivity.*** Because both questionnaires by design produce correlated dimensions, SHAP and single-feature permutation importance redistribute credit among correlated features [42], which can compress or invert within-cluster rankings — most visibly the {sensitivity, complexity} pair in the 16PF, where group-permutation ΔAUC is 0.047 lower than the sum of individual ΔAUCs. As reported in the Results, the overall collinearity is modest and the cluster-level ordering is consistent across models (S1 Table); the formally powered ranking-stability test, on the full trait set, is the cross-configuration concordance of Table 3 (Kendall’s *W* = 0.95 and 0.85 for Big5 and 16PF SHAP, *p* < 0.001). Individual within-cluster rankings should not be over-interpreted.

***Sample composition and generalizability.*** The 16PF cohort (*N* = 44,324) is 2.4 times larger than Big5 (*N* = 18,062); cross-dataset comparisons are mediated by classifier choice and proportionally sized held-out test sets (4,433 vs. 1,807), so the asymmetry does not bias within-dataset estimates; however, it does make the 16PF better powered to detect small effects. We further filter implausible ages (<16 or ≥90) and acquiescence-failed responses but do not partial out age, country, or native-language effects; the cohort is Western-skewed, so predictions in unseen demographics may differ. More fundamentally, the present analysis concerns the internal correspondence between weighting schemes under a fixed covariance structure, rather than population-level prevalence estimates; self-selection bias would primarily affect the magnitude of group differences rather than the rank-order alignment between importance metrics and classical separation measures. Although our results seem to be robust, a further replication with probability-sampled or laboratory-administered data would nonetheless strengthen external validity.

***Data vintage, self-report, and gender measurement.*** The data were collected up to 2014 and are self-reported and unverified, hence subject to social-desirability and acquiescence biases and to item-content sensitivity across the international respondent subset (note, however, we retain only respondents whose acquiescence-check sums fall in the expected range). The prediction target is a self-reported binary gender label (Male / Female) whose pre-2014 conventions conflate biological sex with gender identity; results should be read as predicting that reported binary label rather than gender identity in its modern, nonbinary-inclusive sense.

***Scope of the inductive comparison.*** As developed in the Discussion, our comparison occurs in a dense (N/p=2,955−3,612), additively-linear feature regime; the recovery of established effect sizes is specific to this regime and does directly not generalize to sparse ($\begin{array}{c} p≳N \end{array}$) or nonlinear decision-boundary settings. The present analyses focused on marginal trait-level importance and did not explicitly decompose non-linear interaction contributions; whether such interactions carry unique predictive variance beyond the additive discriminant structure remains an open question. More broadly, although the alignment patterns were robust, the analyses remain correlational and do not imply causal interpretations of trait contributions. Future research should examine whether similar correspondence emerges in other personality frameworks and classification contexts.

## Conclusion

This study examined how model-based feature importance relates to classical measures of gender separation in personality traits. The correspondence between univariate effect size and model-based importance depends on the structural properties of the trait representation. In the Big5, univariate and multivariate rankings converged, whereas in the 16PF, importance rankings aligned more strongly with multivariate discriminant structure than with marginal effect size. These findings indicate that divergence between univariate and model-based importance is consistent with covariance-mediated weighting rather than the emergence of novel structure unique to ML models. Overall, the results confirm that explainability metrics in personality classification can closely track established multivariate geometry and clarify the conditions under which univariate summaries are sufficient versus when multivariate approaches become informative.

## Supporting information

S1 Text Machine learning training and analysis pipeline. This file describes the preprocessing, model training, feature-importance estimation, statistical analysis, seed propagation, and code-to-output mapping used to reproduce the study.
(DOCX)

S1 File Full implementation code. This file contains the complete source code used to reproduce the preprocessing, model training, feature-importance estimation, statistical analysis, seed propagation, and output generation reported in this study.
(ZIP)

S1 Table Complete numerical results underlying the primary and supplementary analyses. This workbook includes full model performance results, complete SHAP and permutation-based trait importance results for Big5 and 16PF, multicollinearity and correlated-feature robustness diagnostics, conditional (group) permutation importance, cluster-level grouped-SHAP concordance, mean- and median-aggregated Spearman correlations, continuous-value correspondence between classical separation measures and model-based importance, LDA-style assumption diagnostics, nonlinear statistical tests, and pooled within-group correlation matrices.
(XLSX)
