# Supplementary material for: Self-reported binary gender prediction from personality traits: Alignment between machine learning importance and classical effect sizes
Source: PLoS One. 2026 Aug 7;21(8):e0355193. doi: 10.1371/journal.pone.0355193 (PMC13450735; doi:10.1371/journal.pone.0355193)
Supplement: Supplementary file 1 [file pone.0355193.s005.pdf]

Dear editor, dear reviewers

We thank you and both reviewers for the constructive and encouraging assessment of our manuscript, "**Self-reported binary gender prediction from personality traits: Alignment between machine learning importance and classical effect sizes**". We were glad that the reviewers found the central idea novel and the analyses technically sound, and we have used their comments to strengthen the manuscript in three ways: (i) a new robustness layer addressing multicollinearity, the LDA assumptions, and continuous (non-rank) importance values; (ii) an explicit delineation of the data regime in which our claims hold, with a new Limitations section; and (iii) a full pass for PLOS ONE style, terminology, and file-naming requirements.

All new analyses are reproducible from the deposited derived data. We have added supplementary tables to the Supporting Information and included nine new references, several of which were published within the last five years. To resolve the upload issues noted in the decision letter, we have consolidated all Supporting Information into a single, comprehensive file. A detailed point-by-point response follows below.

We believe the revisions have substantially strengthened our manuscript and look forward to additional comments (if any).

Sincerely,

Christian Wallraven (for the authors)

Response to Comments of Editor

Please ensure that your manuscript meets PLOS ONE's style requirements, including those for file naming

We thank the editorial office and have carried out a complete style pass over the manuscript:

[Previous] Title

Gender Prediction from Personality Traits: Alignment Between Machine Learning Feature Importance and Classical Multivariate Effect Sizes

[Revised] Title

Self-reported binary gender prediction from personality traits: Alignment between machine learning importance and classical effect sizes

[Added] Author block:

The corresponding-author e-mail line, previously missing, was added (' wallraven@korea.ac.kr'), and the country is now given in full ("Seoul, Republic of Korea").

[Revised] Section and subsection headings

All converted to sentence case (e.g., "Machine Learning Training Framework" → "Machine learning training framework"; "Classification Performance" → "Classification performance").

[Revised] Tables

All table titles were moved above the table grid (Tables 2–4 had them below).

[Revised] Supporting information

The section was moved to the end of the manuscript, after the References. Also, \nameref was adjusted on every citation.

We are unable to open your Supporting Information file. Please kindly revise as necessary and re-upload.

We apologize for the inconvenience. The original Supporting Information had been uploaded as a single `.zip` archive (which the office was unable to open), and its captions still contained template text. We have rebuilt the Supporting Information with complete, final content and now upload it as individual, correctly named files, each of which opens directly:

[S1 Table] — a single multi-part workbook: complete SHAP and permutation-based trait importance results for Big5 and 16PF, multicollinearity and correlated-feature robustness diagnostics, conditional (group) permutation importance, cluster-level grouped-SHAP concordance, mean- and median-aggregated Spearman correlations, continuous-value correspondence between classical separation measures and model-based importance, LDA-style assumption diagnostics, nonlinear statistical tests, and pooled within-group correlation matrices.

[S1 Text] — the machine-learning training and analysis pipeline.

[S1 File] — the reproducibility code package.

If the reviewer comments include a recommendation to cite specific previously published works, please review and evaluate these publications to determine whether they are relevant and should be cited. There is no requirement to cite these works unless the editor has indicated otherwise.

We thank you for this guidance. Reviewer 2 explicitly invoked **Wolpert's (1996) "No Free Lunch" theorem**. We reviewed and evaluated it, found it directly relevant to the inductive-scope argument, and now cite it in the new scope-delineation paragraph. Beyond this directly recommended work, in implementing the additional analyses requested by the reviewers, we evaluated and incorporated appropriate recent methodological literature, citing each at its point of use. Specifically, in support of Reviewer 1's analyses regarding **multicollinearity and correlated-feature robustness**, we have included **Aas, Jullum & Løland (2021)**, **Jullum, Redelmeier & Aas (2021)**, **Debeer & Strobl (2020)**, and **Chamma, Engemann & Thirion (2023)**. Each work was assessed for its direct relevance prior to inclusion, in accordance with editorial guidance.

### Editor comments

The manuscript title and main text frequently use "gender," while many of the references and discussion refer to "sex differences." In fact, the data variable is a self-reported

binary male/female category. I suggest using “self-reported binary gender” consistently throughout the manuscript.

We thank you for this observation, with which we fully agree; the target variable is indeed a self-reported binary Male/Female item. We now use "self-reported binary gender" as the defined target term — introduced in the Title and Abstract and used in the Introduction, Methods, and Limitations — and refer to it as "gender" consistently in the remaining narrative. Critically, the previously mixed "sex differences" wording no longer appears anywhere in our own text. Rather than insert a separate definitional statement, we defined the term within the existing Datasets sentence:

[Previous] Title

> Gender Prediction from Personality Traits: Alignment Between Machine Learning Feature Importance and Classical Multivariate Effect Sizes

[Revised]

> Self-reported binary gender prediction from personality traits: Alignment between machine learning importance and classical effect sizes

[Previous] Dataset

> From this resource, we selected two widely used personality instruments that provide both personality measures and self-reported gender labels: the Big5 and the 16PF.

[Revised] Dataset (Line 246-247)

> From this resource, we selected two widely used personality instruments that provide both personality measures and a self-reported binary gender label (Male / Female): Big5 and the 16PF. We distinguish this variable from biological sex and from contemporary multi-category gender constructs.

Please use references from the past five years.

We have substantially strengthened the recent literature base with several references from recent years added and discussed (see below).

[Added]

> **- Hofmann et al. (2025, Journal of Research in Personality)**
> - **Rammstedt et al. (2026, Personality and Individual Differences)**

- **Eagly & Revelle (2022, Perspectives on Psychological Science)**
- **Del Giudice (2022, Springer; 2024, Behavior Research Methods)**

After revising the full manuscript, please read it through carefully to check for any potential errors.

We carried out another full proofreading pass — checking terminology, every cross-reference, and every reported number against the deposited derived data, including the figures — and corrected the following:

[Revised] Recomputed values

> **Table 1:** Agreeableness Cronbach's α corrected from 0.727 to **0.831**
>
> **Discussion** (non-linear robustness): the non-linear-only permutation alignment was corrected from ρ = 0.829 to **0.828**.

[Revised] Statistical-method consistency

> **Table 4 and Fig 1:** Because the asymptotic (t-approximation) Spearman p-value is unreliable at N = 5, we report exact permutation p-values for the Big5, as stated in the Methods. The Big5 significance markers in Table 4 were corrected from `**` to `*` (exact p = 0.017–0.033 at N = 5), and the Fig 1 legend now reports the exact values (SHAP p = 0.033, Perm p = 0.017). The 16PF (N = 15) is unaffected.

[Revised] Figure corrections found in the re-read:

> **Fig 1:** We corrected a labeling error on the Big Five axis, where Extraversion and Conscientiousness had been transposed on the Cohen's |d| rank axis (Extraversion |d| = 0.13, Conscientiousness |d| = 0.08). The affected data points now fall on the identity line, consistent with the reported ρ.
>
> **Fig 2:** The importance axis was recomputed on the same mean-of-rank consensus used in Table 4 (previously a rank-of-mean variant), so the plotted points now correspond exactly to the reported from ρ = 0.846 in Big5 and 0.826 in 16PF.

We further confirmed that every Table, Figure, and Supporting-Information cross-reference resolves, that all figures plot the same mean-of-rank consensus

reported in Table 4, and that the Abstract, Results, and Supporting Information report identical values.

Response to Comments of Reviewer 1

MULTICOLLINEARITY: To make the analysis more robust, given that personality traits are correlated a priori at the theoretical level and that the analyses here use SHAP and permutation, which are unstable with correlated constructs, it is necessary to directly add a multicollinearity analysis to the paper using the correlation matrix (since both SHAP and permutation importance are sensitive) and possibly add conditional permutation and SHAP as a robustness check.

We would like to thank you for this important comment. We agree that, because personality traits are correlated a priori and SHAP and single-feature permutation importance are sensitive to correlated constructs, a direct multicollinearity analysis is needed. In response, we added correlation-matrix diagnostics together with conditional (group) permutation importance and grouped SHAP as robustness checks, as follows:

[ADDED] Methods new subsection: "Multicollinearity diagnostics" [Line223-233]:

> Because SHAP and single-feature permutation importance are sensitive to correlated predictors, we quantified inter-trait dependence for each trait feature set (max/median |r|, condition number and Belsley condition index, eigenvalue-entropy effective dimensionality, and variance inflation factor; S1 Table). As robustness checks we additionally computed conditional (group) permutation importance and cluster-level grouped SHAP: traits were hierarchically clustered by Ward linkage on the Spearman correlation distance $d = 1 - |r_s|$, the dendrogram was cut at $d = 0.7$, each cluster was permuted jointly to preserve within-cluster correlation, and mean(|SHAP|) was summed within clusters.

[ADDED] Results in "Importance stability across configurations" [Line265-272]:

> Inter-trait correlations were modest in both feature sets (maximum $|r| = 0.34$ in the Big5 and 0.75 in the 16PF; maximum variance inflation factor 1.22 and 3.35; Belsley condition index 1.72 and 4.58, both below the standard threshold of 10; S1 Table). Conditional (group) permutation importance and cluster-level grouped

> SHAP confirmed that, although SHAP and single-feature permutation redistribute credit within correlated clusters, the cluster-level importance ordering remained fully consistent across the five classifiers (S1 Table). The reported trait-importance rankings are therefore not artefacts of collinearity.

[ADDED] S1 Table (diagnostics, conditional permutation, grouped SHAP):

> in the Supporting Information; the most visible redundancy is the {sensitivity, complexity} pair in the 16PF (group ΔAUC 0.047 below the sum of individual ΔAUCs).

LDA: While not strictly necessary, it would be more robust to add an analysis of the basic applicability assumptions (covariance and distribution), i.e., adding analyses of equal covariance across groups and the data distribution.

We would like to thank you for this comment. We agree that reporting the basic LDA applicability assumptions makes the analysis more robust. In response, we added formal equal-covariance and distribution diagnostics (S1 Table) and clarified that LDA is used only to obtain discriminant weights, not as the classifier, as follows:

[ADDED] Results in “Alignment with classical separation measures” (Line172-177):

> Classification itself relies on LR—whose asymptotic justification requires only that the conditional log-odds be linear in the features, not multivariate normality—together with four nonlinear, distribution-free classifiers. Formal multivariate-normality and equal-covariance diagnostics (Box’s M, Mardia, Henze–Zirkler) are therefore reported in S1 Table as a transparency check rather than as validation of the inference engine.

RANKING: Although the use of rankings is justified given the different scales, it would be appropriate to add complementary analyses on normalized continuous values.

We would like to thank you for this comment. We agree that, although ranking is justified by the differing scales, a complementary analysis on normalized continuous values strengthens the result. In response, we added an L1-normalized continuous-value analysis (new Table 5; per-cell values in S3 Table), as follows:

[ADDED] Result (Line 293 – 302):

Because rank correlations discard magnitude, we repeated the alignment analysis on L1-normalized continuous values: within each (model × resampling) cell we L1-normalized the per-trait |d|, |w̃|, mean(|SHAP|), and permutation importance and computed Pearson r and top-5 Jaccard overlap (Table 5; per-cell values in S3 Table). The continuous-value correlations are uniformly at least as high as the rank-based values of Table 4, confirming the rank conversion was conservative rather than optimistic. Critically, replacing the univariate |d| with the multivariate discriminant weight |w̃| restores near-perfect correspondence for the 16PF (median Pearson r = 0.987 for SHAP, 0.940 for permutation), indicating that the |d|–importance gap in the 16PF reflects the inter-trait correlation structure the model uses correctly, not a misreading of the data.

ADD A PARAGRAPH ON LIMITATIONS: It is strongly necessary to add a paragraph on limitations or include them in the conclusions (but giving them appropriate space), highlighting in more detail all critical aspects, such as the presence of correlations, the size difference between Big 5 and 16pf, data dated 2014, common self-report issues such as social desirability, distinction between sex and gender (binary classification only), sensitivity of model importances , and no control for age, origin, or other factors.

We would like to thank you for this comment. We agree that the limitations deserve dedicated space. In response, we added a stand-alone "Limitations" section before the Conclusion, organized into four labeled points that together cover every item you listed, as follows:

[Previous] Discussion

Several limitations warrant consideration. Both datasets were based on self-report instruments and collected from self-selected volunteers on an open online platform with predominantly Western respondents. However, the present analysis concerns the internal correspondence between weighting schemes under a fixed covariance structure, rather than population-level prevalence estimates; self-selection bias would primarily affect the magnitude of group differences rather than the rank-order alignment between importance metrics and classical separation measures. Replication with probability-sampled or laboratory-administered data would nonetheless strengthen external validity. Although alignment patterns were robust, the analyses remain correlational and do not imply causal interpretations of trait contributions. The present analyses focused on marginal trait-level importance and did not explicitly decompose

non-linear interaction contributions; whether such interactions carry unique predictive variance beyond the additive discriminant structure remains an open question. Future research should examine whether similar correspondence emerges in other personality frameworks and classification contexts.

[Revised]: Limitation [Line 419-461]

Several constraints qualify the scope of our findings.

Inter-trait correlations and importance sensitivity. Because both questionnaires by design produce correlated dimensions, SHAP and single-feature permutation importance redistribute credit among correlated features [42], which can compress or invert within-cluster rankings — most visibly the {sensitivity, complexity} pair in the 16PF, where group-permutation ΔAUC is 0.047 lower than the sum of individual ΔAUCs. As reported in the Results, the overall collinearity is modest and the cluster-level ordering is consistent across models (S1 Table); the formally powered ranking-stability test, on the full trait set, is the cross-configuration concordance of Table 3 (Kendall's W = 0.95 and 0.85 for Big5 and 16PF SHAP, $p < 0.001$). Individual within-cluster rankings should not be over-interpreted.

Sample composition and generalizability. The 16PF cohort (N = 44,324) is 2.4 times larger than Big5 (N = 18,062); cross-dataset comparisons are mediated by classifier choice and proportionally sized held-out test sets (4,433 vs. 1,807), so the asymmetry does not bias within-dataset estimates; however, it does make the 16PF better powered to detect small effects. We further filter implausible ages (< 16 or ≥ 90) and acquiescence-failed responses but do not partial out age, country, or native-language effects; the cohort is Western-skewed, so predictions in unseen demographics may differ. More fundamentally, the present analysis concerns the internal correspondence between weighting schemes under a fixed covariance structure, rather than population-level prevalence estimates; self-selection bias would primarily affect the magnitude of group differences rather than the rank-order alignment between importance metrics and classical separation measures. Although our results seem to be robust, a further replication with probability-sampled or laboratory-administered data would nonetheless strengthen external validity.

Data vintage, self-report, and gender measurement. The data were collected up to 2014 and are self-reported and unverified, hence subject to social-desirability and acquiescence biases and to item-content sensitivity across the international respondent subset (note, however, that we retain only respondents whose

acquiescence-check sums fall in the expected range). The prediction target is a self-reported binary gender label (Male / Female) whose pre-2014 conventions conflate biological sex with gender identity; results should be read as predicting that reported binary label rather than gender identity in its modern, nonbinary-inclusive sense.

Scope of the inductive comparison. As developed in the Discussion, our comparison occurs in a dense (N/p = 295–3,612), additively-linear feature regime; the recovery of established effect sizes is specific to this regime and does not directly generalize to sparse ($p \gtrsim N$) or nonlinear decision-boundary settings. The present analyses focused on marginal trait-level importance and did not explicitly decompose non-linear interaction contributions; whether such interactions carry unique predictive variance beyond the additive discriminant structure remains an open question. More broadly, although the alignment patterns were robust, the analyses remain correlational and do not imply causal interpretations of trait contributions. Future research should examine whether similar correspondence emerges in other personality frameworks and classification contexts.

Response to Comments of Reviewer 2

A foundational premise in machine learning is that in sparse data regimes, where dimensionality outnumbers observed data and unbiased models would otherwise fail, the appropriate choice of an inductive bias can enable successful interpolation of unobserved outcomes. This has led to the application of machine learning methods by researchers hoping to discover hidden structure in their own data, often with little attention to the particular choice of inductive bias. However, Wolpert's (1996, Neural Computation) famous "No Free Lunch" theorem proves that there is no universally optimal learning algorithm across learning objectives, and thus matching the learning algorithm's inductive bias with the learning objective becomes essential. Cho et al. invite the question of whether particular inductive biases are needed at all, but they spend too little time delimiting the scope of their study. This is fine for sophisticated readers who understand the underlying machine learning theory, but places non-specialist readers at risk of seriously misunderstanding the findings.

This study focuses on a particular kind of data in a particular inductive regime where data exceeds dimensionality by four orders of magnitude. Further, the personality trait data in question is by design intended to provide an additive linear factorization of personality variance, which induces a particular data structure. Its geometry is linear in all parameters with topology devoid of intrinsic "holes", i.e. all combinations of parameters are plausibly observable and there is no configuration that is a priori precluded. In this regime bias-variance tradeoffs are largely moot due to plentiful data, and nonlinear methods offer little advantage. Indeed, the performance Cho et al report for their linear models are nearly indistinguishable from their nonlinear models (Table 2, e.g. logistic regression of gender Big5 yields AUC=0.678 vs. random forests which yield

0.679; note that AUC is the area under the receiver operator characteristic curve and can be interpreted as classification accuracy in a binary forced choice test). Consequently, their conclusion that feature importance in machine learning models is highly correlated with linear statistical metrics like Cohen's d and Mahalanobis distance is essentially anticipated by the data regime, feature space, and learning objective.

We would like to thank you for this framing, which we fully accept. In response, we added a scope-delineation paragraph at the head of the Discussion that addresses both points at once — it states the data-rich, additively-linear regime in which the learner's inductive bias carries little weight, reports the near-identical linear/nonlinear AUCs, and concludes that the correspondence is anticipated by the regime and therefore does not license generalization to sparse or nonlinear settings:

[ADDED] Discussion (Line 316 – 332):

> The correspondence we report between feature importance and linear statistical metrics is partly a consequence of our data regime. Both datasets satisfy a large observations-per-feature ratio at the trait level used here — N/p = 3,612 (Big5) and 2,955 (16PF) — more than three orders of magnitude above unity, well into the data-rich regime in which the inductive bias of the learning algorithm carries less weight [43]. Moreover, the Big5 and 16PF are by design additive linear factorisations of personality variance, so the discriminant boundary in this basis is well-approximated by a hyperplane; this is confirmed by the near-identical test AUCs of LR and the tree-/boosting-based models (0.678 for LR vs. 0.679 for RF in the Big5 trait set, and 0.790 vs. 0.798 for the 16PF trait set; Table 2). Our finding that machine-learning feature importance is highly correlated with Cohen's d, the standardized discriminant weight $\tilde{w}$, and Mahalanobis distance is therefore partly anticipated by the dense, linear nature of the problem; it does not entitle a generalization to sparse feature regimes ($p \gtrsim N$) or to nonlinear decision-boundary problems such as those arising from digital footprints or behavioural traces. The mechanisms we describe — credit redistribution among correlated traits, suppressor effects, multivariate vs. univariate divergence — are likely to translate to those settings, but the specific quantitative correspondence between importance and effect size is not.

The authors admit in their discussion that the "topology of inter-trait correlations" is a critical factor underlying their findings but spend no time describing what they mean by this. Further, in their introduction they motivate their interest in machine learning based predictions from personality measures by invoking "digital footprints and behavioral

traces", a data regime with differing dimensionality and topology than purpose made research instruments like Big5 and PF16 traits. This leaves non-specialist readers at risk of misunderstanding the scope of their findings and mistakenly generalizing from findings based on a densely sampled linear discrimination boundary to sparsely sampled nonlinear boundaries. The authors' discussion should deliberately engage with this problem and scope the limits of their findings at least with respect to the kinds of applications they cite for motivation in their introduction.

We would like to thank you for this comment. In response we (i) define what we mean by "topology," and (ii) rescope the introductory digital-footprint motivation.

First, we replaced the previously undefined "topology" with an explicit definition tied to the suppressor mechanism:

[Previous] Discussion

> The critical factor is therefore not dimensionality alone, but the strength and topology of inter-trait correlations.

[Revised] Discussion (Line 367 – 371)

> The decisive factor is therefore not dimensionality, nor even the overall strength of the correlations, but their topology — which traits covary, and with what sign — since this configuration is what R−1_pooled propagates into the up- and down-weighting of traits and the emergence.

Second, we inserted a scoping sentence after the digital-footprint motivation in the Introduction (the motivation itself is unchanged):

[Added] Introduction (Line 29-33)

> Those settings are the targeted application domain of importance-based interpretability; the present work operates one step earlier, validating under a controlled, additively factorized feature design that feature importance does in fact recover established statistical effects before such methods are transported to sparse, behavioural-trace regimes.

The abstract would benefit from clarifying that both linear and nonlinear machine learning models were trained. This can be as simple as changing "We trained multiple machine learning models" to "We trained multiple linear and nonlinear machine learning models".

We would like to thank you for this comment. In response, we revised the abstract as follow:

[Previous] Abstract

> We trained multiple machine learning models to predict gender from trait scores and derived feature-importance rankings using SHAP and permutation importance.

[Revised] Abstract

> We trained multiple linear and nonlinear machine learning models (Logistic Regression, Random Forest, Extra Trees, LightGBM, and Explainable Boosting Machine) to predict self-reported binary gender from trait scores and derived feature-importance rankings using SHAP and permutation importance.

At line 225, recontextualize the resampling for the reader. Instead of writing "Resampling effects were negligible in the 16PF..." Write something like "Choice of resampling method to correct for class imbalance had a negligible effect on model accuracies in the 16PF…". This will help the reader remember what resampling was for after having last read about it several paragraphs prior in the methods section.

We would like to thank you for this comment. In response, we recontextualized the resampling sentence in the Results as follows:

[Previous]

> Resampling effects were negligible in the 16PF

[Revised] Results (Line 246-247)

> Choice of resampling method to correct for class imbalance had a negligible effect on model accuracies in the 16PF

Lines 239-241 and 249-250 appear to contradict themselves. In the former it sounds like feature ranks are averaged across models and then correlated with statistical dispersion measures (Cohen's d, Mahalanobis distance, etc.), while in the latter it sounds like feature ranks are correlated with statistical dispersion measures independently for each model class and then correlations are averaged. Based on the methods write up, I believe the authors did the latter. The language should be homogenized to avoid confusion.

We would like to thank you for catching this. On re-examining the implementation, the reported correlations (Table 4, Figs 1–2) are obtained by **aggregating the per-trait**

importance ranks across the 20 configurations into a single consensus ranking, then computing one Spearman correlation against each classical measure (the "former" reading; this is what the analysis code computes, §5.1). The contradiction arose because one passage described this aggregate-then-correlate procedure and another a per-configuration-then-average procedure. In response, we homogenized all wording (Methods, Results, Table 4 caption) to the implemented procedure — noting that per-configuration averaging gives the same conclusions (robustness check) — as follows:

[Previous] Method

> These values, together with |di|, | ˜wi|, and |ci|, were converted to trait-level ranks, and Spearman's ρ was computed between the classical and model-based rankings within each configuration and averaged across configurations.

[Revised] Method (Line 218 - 221)

> These values, together with |di|, | ˜wi|, and |ci| were converted to trait-level ranks ; the model-based ranks were then aggregated across configurations into a single consensus ranking, and Spearman's ρ was computed between this aggregated ranking and each classical ranking.

[Previous] Result

> Spearman correlations were computed within each configuration and averaged across configurations (Table 4); configuration-level correlations and median-aggregated results are reported in S1 Table.

[Revised] Result (Line 279 - 282)

> For each importance measure, trait rankings were aggregated across the 20 configurations and a single Spearman correlation was computed against each classical measure (Table 4); the median-aggregated and per-configuration variants are consistent, which are reported in S1 Table.

The claim on lines 316-318 that "the empirical result confirms that this relationship extends … to nonlinear classifiers" is too strong. The empirical results only confirm that this relationship extends to nonlinear classifiers when applied to data with linear discriminant boundaries.

We would like to thank you for this comment. In response, we qualified the claim exactly as you suggest — restricting it to data with (near-)linear discriminant boundaries and explicitly excluding genuinely nonlinear problems — as follows:

[Previous] Discussion

> The empirical result confirms that this relationship extends, in attenuated form, to non-linear classifiers (see below).

[Revised] Discussion (Line 382-386)

> The empirical result indicates that this relationship extends, in attenuated form, to the non-linear classifiers examined here only because they are applied to data with a near-linear discriminant boundary, as in the present additively linear feature spaces; it does not establish that the correspondence holds for genuinely nonlinear decision-boundary problems (see below).
