# Supplementary material for: Self-reported binary gender prediction from personality traits: Alignment between machine learning importance and classical effect sizes
Source: PLoS One. 2026 Aug 7;21(8):e0355193. doi: 10.1371/journal.pone.0355193 (PMC13450735; doi:10.1371/journal.pone.0355193)
Supplement: Supplementary file 2 [file pone.0355193.s001.docx]

# S1 Text. Machine learning training and analysis pipeline

This file describes the preprocessing, model training, feature-importance estimation, statistical analysis, seed propagation, and code-to-output mapping used to reproduce the study.

## Software environment

All analyses were implemented in Python 3.8. The required packages and their versions are listed below:

| Package | Version | Role |
| --- | --- | --- |
| numpy | 1.21.6 | Numerical computation |
| pandas | 1.3.5 | Data manipulation |
| scikit-learn | 0.23.2 | Model training, cross-validation, evaluation |
| scipy | 1.5.4 | Statistical tests (Friedman, Spearman, t-test) |
| imbalanced-learn | 0.7.0 | Resampling (SMOTE, Tomek Links, SMOTETomek) |
| lightgbm | 3.3.2 | LightGBM classifier |
| interpret | 0.2.7 | Explainable Boosting Machine |
| shap | 0.41.0 | SHAP value computation |
| matplotlib | 3.5.1 | Figure generation |
| seaborn | 0.11.2 | Figure generation |
| joblib | 1.0.1 | Model serialization |

The complete environment specification is provided in environment.yml and can be installed via conda env create -f environment.yml. To minimize numerical variation across systems, using the provided conda environment is recommended when reproducing results.

## Input data

The reproducibility package includes two pre-processed datasets derived from the Open-Source Psychometrics Project(https://openpsychometrics.org/_rawdata/):

- **Big Five Inventory (Big5):** 18,062 respondents scored on 5 trait scales (Extraversion, Agreeableness, Conscientiousness, Neuroticism, Openness), each computed from 10 items (50 items total).
- **16 Personality Factor (16PF):** 44,324 respondents scored on 15 trait scales (warmth, emotional stability, assertiveness, gregariousness, dutifulness, friendliness, sensitivity, distrust, imagination, reserve, anxiety, complexity, introversion, orderliness, emotionality), each computed from 10 items (150 items total). Consistent with prior large-scale analyses using the same data source, one primary factor was excluded due to measurement limitations in the online questionnaire, resulting in a final set of 15 personality traits used for analysis.

### Data cleaning

Following established large-scale analyses of gender differences in personality, respondents were excluded if they met any of the following criteria: (1) 25 or more missing responses, (2) uniform responding across all items (e.g., selecting only the minimum or maximum scale value), or (3) self-reported age outside the range of 16 to 90 years. Because this study focused on binary gender classification, only participants who self-identified as male or female were retained; records with other or unspecified gender categories were excluded due to limited representation. For the 16PF dataset, an additional response accuracy index was available; participants with invalid accuracy scores were excluded, and only those with an accuracy score exceeding 50% were retained, consistent with prior recommendations. After cleaning, the final sample comprised 44,324 participants in the 16PF (59.6% female) and 18,062 participants in the Big5 (60.7% female).

The retained binary gender label was encoded as 1 = male and 2 = female. The processed data files (data/big5_processed.csv, data/pf16_processed.csv) contain trait scores, individual item responses, and gender.

## Pipeline overview

The full pipeline is executed through run_all.sh and consists of six phases:

0. **Data preprocessing** (preprocess.py; runs only if raw data is present in data/raw/),
1. **Model training** (train.py),
2. **Feature explanation and importance estimation** (explain.py),
3. **Aggregation of model outputs** (analyze_results.py),
4. **Statistical analysis** (stats_analysis.py), and
5. **Figure generation** (plot_results.py).

Phase 0 converts the raw survey files into the processed CSVs used by subsequent phases; if the processed files are already present and raw data is not provided, this phase is skipped automatically. Phase 1 trains 40 models in total, corresponding to 5 classifiers × 4 resampling conditions × 2 datasets (20 configurations per dataset). Phase 2 computes permutation importance and SHAP-based importance measures for each trained model. Phase 3 aggregates per-model outputs into summary analysis files per dataset. Phase 4 generates the statistical tables reported in the manuscript and supporting information. Phase 5 produces the final figures.

## Model training and resampling

### Data splitting

For each of the 40 model configurations, the data were split into training (90%) and test (10%) sets using stratified random sampling to preserve the gender ratio.

### Classifiers

The five evaluated classifiers were:

| Abbreviation | Classifier | Hyperparameter tuning |
| --- | --- | --- |
| lr | Logistic Regression | RandomizedSearchCV (50 iterations) |
| rf | Random Forest | RandomizedSearchCV (50 iterations) |
| et | Extra Trees | RandomizedSearchCV (50 iterations) |
| lightgbm | LightGBM | RandomizedSearchCV (50 iterations) |
| ebm | Explainable Boosting Machine | Default settings (interactions = 0) |

### Hyperparameter search spaces

- **Logistic Regression:** C ∈ logspace(−3, 3, 50 values), penalty ∈ {L1, L2}, solver = saga.
- **Random Forest:** n_estimators ∈ {100, 200, 300}, max_depth ∈ {5, 10, 15, 20, None}, min_samples_split ∈ {2, 5, 10}, min_samples_leaf ∈ {1, 2, 4}.
- **Extra Trees:** Same search space as Random Forest.
- **LightGBM:** n_estimators ∈ {100, 200, 300}, max_depth ∈ {3, 5, 7, 10}, learning_rate ∈ {0.01, 0.05, 0.1}, num_leaves ∈ {15, 31, 63}, min_child_samples ∈ {10, 20, 30}.
- **Explainable Boosting Machine:** No hyperparameter search; used with default parameters and interactions fixed to zero.

### Training procedure

Each model was trained using an imbalanced-learn pipeline that chains (optionally) a resampler, a standard scaler, and the classifier. All models were evaluated internally via 10-fold stratified cross-validation within the training data. Within each fold, resampling and z-score normalization were fitted on the training subset only, followed by model fitting; validation predictions were generated using normalization parameters derived from the corresponding training fold, and validation data were not resampled.

For LR, RF, ET, and LightGBM, this 10-fold CV was embedded within RandomizedSearchCV to select the best hyperparameter configuration from 50 random candidate combinations (500 total model fits per model–resampling combination), using AUC as the scoring metric. For EBM, which was used with default parameters (interactions = 0), no hyperparameter search was performed, but the same fold-wise preprocessing and cross-validation framework was applied to estimate internal performance.

After cross-validation, the final pipeline was refit on the full training set using the selected (or default) parameters with the same resampling and normalization procedure. Final evaluation was conducted on the held-out test set, where normalization parameters estimated from the training data were applied without resampling.

### Resampling

Four resampling conditions were evaluated:

| Condition | Method |
| --- | --- |
| None | No resampling |
| SMOTE | Synthetic Minority Over-sampling Technique |
| TomekLinks | Tomek Links under-sampling |
| SMOTETomek | Combined SMOTE + Tomek Links |

Resampling was applied inside the cross-validation pipeline (via imbalanced-learn’s Pipeline) to prevent data leakage. That is, resampling was applied only to the training folds and never to the validation or test folds.

### Evaluation metrics

Given the moderate class imbalance present in both datasets, predictive performance was primarily evaluated using AUC (area under the ROC curve). Accuracy and F1 score (macro-averaged) were reported as secondary metrics. Additional metrics (precision, recall) were retained in per-model output files.

## Feature importance and SHAP analysis

Feature importance was estimated using two complementary approaches:

### Permutation importance

Permutation importance quantifies the decrease in test-set AUC after permuting each trait, capturing its contribution to predictive performance. It was computed on the held-out test set using scikit-learn’s permutation_importance with AUC as the scoring metric and 30 repeats. The random state was propagated from the pipeline seed.

### SHAP values

SHAP (SHapley Additive exPlanations) values were computed using model-appropriate explainers:

- **Tree-based models (RF, ET, LightGBM):** shap.TreeExplainer (exact TreeSHAP algorithm; deterministic).
- **Logistic Regression:** shap.LinearExplainer (exact; deterministic).
- **Explainable Boosting Machine:** shap.Explainer (model-agnostic permutation-based explainer; seeded for reproducibility).

All feature importance analyses were conducted exclusively on held-out test data, using the preprocessed representations produced by the fitted pipeline. For binary classification, SHAP values for the positive class (Female) were used: positive SHAP values indicate a push toward the Female prediction, and negative values indicate a push toward Male.

### Aggregation across configurations

For each of the 20 model configurations within each dataset, features were ranked by mean absolute SHAP value and by permutation importance. Feature importance rankings reported in the manuscript (Table 3) represent mean and median ranks across all 20 configurations within each dataset. Mean aggregation was used in the primary analysis, and median aggregation was computed as a robustness check.

## Statistical analysis and outputs

The stats_analysis.py script generates all manuscript tables and supplementary outputs.

### Table 1: Descriptive statistics and Cohen’s d

Gender differences in personality traits were quantified using Cohen’s d with pooled standard deviation. The sign convention is d > 0 for Female > Male. The output file includes Cohen’s d values and their associated 95% confidence intervals. Internal consistency for each trait scale was assessed using Cronbach’s alpha, computed from the constituent items with appropriate reverse scoring.

### Table 2: Model performance

Model performance was summarized per model type by averaging test-set metrics across the four resampling conditions (mean ± SD). The full results across all 40 runs (20 configurations per dataset) were also saved.

### Table 3: Feature importance rankings

Features were ranked within each of the 20 configurations per dataset by mean absolute SHAP value and by permutation importance. Mean and median ranks across configurations are reported.

Cross-configuration concordance was assessed using Kendall’s coefficient of concordance (W). Traits were treated as ranked items and each model–resampling combination as an independent rater.

Alignment between classical and ML-based measures was assessed using Spearman’s rank correlation (ρ) between rankings derived from |d_i|, |w̃_i|, and |c_i|, and rankings derived from SHAP and permutation importance. For each configuration, SHAP importance was computed as the mean absolute SHAP value per trait on the held-out test set, and permutation importance as the decrease in test-set AUC after permuting each trait. Correlations were computed within each configuration and averaged across configurations. Mean aggregation was used in the primary analysis, with median aggregation reported as a robustness check. For the Big5 dataset (N = 5 traits), exact permutation p-values for Spearman’s ρ were computed by exhaustive enumeration of all 5! = 120 permutations, since the t-distribution approximation is unreliable at small N.

### Table 4: Multivariate analysis

Multivariate gender differences were analyzed using discriminant analysis weights derived from the pooled within-group correlation matrix. For each dataset, the pooled within-group covariance matrix Σ_pooled was computed as ((n_m − 1)·Σ_m + (n_f − 1)·Σ_f) / (n_m + n_f − 2), then converted to a correlation matrix R_pooled. Standardized discriminant function weights were computed as w̃ = R_pooled⁻¹·d, where d is the vector of Cohen’s d values. Mahalanobis’ D² was computed as d’·w̃. Traits where the product d·w̃ < 0 were flagged as exhibiting suppressor effects.

### Friedman tests (model comparison)

Friedman tests on test-set AUC were conducted separately for each dataset. Two factors were tested independently: (1) model effect, with model type as the main factor pooling results across resampling conditions, and (2) resampling effect, with resampling method as the main factor pooling results across model types. Kendall’s W was computed as an effect-size measure for each test.

### Non-linear robustness checks

Kendall’s W and Spearman ρ analyses were repeated using only the 16 non-linear model configurations per dataset (excluding Logistic Regression) to assess whether importance rankings were robust to the exclusion of the linear classifier.

### Multicollinearity and correlated-feature robustness

Because the trait features are intercorrelated, several diagnostics were computed on the trait feature sets (Big5, 5 features; 16PF, 15 features) and reported in S1 Table.

**Dependence diagnostics**: For each feature set, we computed the maximum, median, and mean absolute pairwise Pearson correlation; the condition number κ₂(R) and Belsley condition index (√κ₂) of the correlation matrix; the eigenvalue-entropy effective dimensionality; and the maximum and median variance inflation factor (VIF, via statsmodels). Correlated features were grouped by hierarchical (Ward-linkage) clustering on the Spearman distance 1 − |r| at a 0.7 cut.

**Conditional (group) permutation importance**: For each correlated cluster, all member features were permuted jointly, and the resulting decrease in test-set AUC (group-permutation ΔAUC) was compared with the sum of the single-feature ΔAUC values; the gap (group minus sum), averaged across the five classifiers, indexes redundancy (negative) versus synergy (positive) within the cluster.

**Cluster-level grouped-SHAP concordance**: Per cluster, mean absolute SHAP values were summed across member features, and the cross-classifier agreement of these cluster-level scores was quantified by pairwise Spearman ρ.

### Distributional (LDA) assumption diagnostics

To accompany the discriminant-analysis weights (Table 4), formal multivariate diagnostics were computed by gender group: Box's M test of equal covariance, and Mardia's and the Henze–Zirkler tests of multivariate normality, together with per-feature Shapiro–Wilk statistics, skewness, and excess kurtosis (via pingouin). These are reported as transparency checks; classification relies on logistic regression and four distribution-free non-linear classifiers, not on linear discriminant analysis.

### Continuous-value correspondence

To complement the rank-based alignment, the analysis was repeated on L1-normalised continuous values: within each (model × resampling) configuration, the per-trait |d|, |w̃|, mean absolute SHAP, and permutation importance were L1-normalised, and the Pearson correlation and top-5 Jaccard overlap between each effect-size measure and each importance measure were computed and summarised (median across the 20 configurations).

### Supplementary outputs

The statistical outputs underlying the main and supplementary analyses were consolidated into a single supplementary workbook (S1 Table). This includes full model performance results across all 40 runs, complete SHAP and permutation-based trait importance results, mean- and median-aggregated Spearman correlations, non-linear robustness tests, the multicollinearity diagnostics, the conditional (group) permutation importance, the cluster-level grouped-SHAP concordance, the LDA-style assumption diagnostics, the continuous-value (L1-normalised) correspondence, and the pooled within-group correlation matrices for Big5 (5 × 5) and 16PF (15 × 15).

## Seed-controlled reproducibility

A single --seed argument (default: 42) is propagated from run_all.sh to all stochastic procedures:

| Procedure | Seed usage |
| --- | --- |
| Train/test split | random_state in train_test_split |
| Cross-validation folds | random_state in StratifiedKFold |
| Randomized hyperparameter search | random_state in RandomizedSearchCV |
| Model initialization | random_state in each classifier constructor |
| SMOTE / SMOTETomek resampling | random_state in resampler constructor |
| Permutation importance | random_state in permutation_importance |
| SHAP (EBM only) | seed parameter in shap.Explainer |
| Global random state | random.seed(), np.random.seed(), PYTHONHASHSEED |

Deterministic phases—aggregation (Phase 3), statistical analysis (Phase 4), and figure generation (Phase 5)—do not depend on the seed. Minor floating-point differences may occur across hardware architectures or library versions, but these do not affect rankings or substantive conclusions.
