# Supplementary material for: Self-reported binary gender prediction from personality traits: Alignment between machine learning importance and classical effect sizes
Source: PLoS One. 2026 Aug 7;21(8):e0355193. doi: 10.1371/journal.pone.0355193 (PMC13450735; doi:10.1371/journal.pone.0355193)
Supplement: Peer review history

# Peer review: Decision Letter 0

**DOI:** 10.1371/journal.pone.0355193.r001

**Authors:** Chong Liu

14 May 2026

PONE-D-26-12774

Gender Prediction from Personality Traits: Alignment Between Machine Learning Feature Importance and Classical Multivariate Effect Sizes

PLOS One

Dear Dr. Wallraven,

Thank you for submitting your manuscript to PLOS ONE. After careful consideration, we feel that it has merit but does not fully meet PLOS ONE’s publication criteria as it currently stands. Therefore, we invite you to submit a revised version of the manuscript that addresses the points raised during the review process.

Please submit your revised manuscript by Jun 28 2026 11:59PM. If you will need more time than this to complete your revisions, please reply to this message or contact the journal office at plosone@plos.org. When you're ready to submit your revision, log on to https://www.editorialmanager.com/pone/ and select the 'Submissions Needing Revision' folder to locate your manuscript file.

Please include the following items when submitting your revised manuscript:

- A letter that responds to each point raised by the academic editor and reviewer(s). You should upload this letter as a separate file labeled 'Response to Reviewers'.
- A marked-up copy of your manuscript that highlights changes made to the original version. You should upload this as a separate file labeled 'Revised Manuscript with Track Changes'.
- An unmarked version of your revised paper without tracked changes. You should upload this as a separate file labeled 'Manuscript'.

If you would like to make changes to your financial disclosure, please include your updated statement in your cover letter. Guidelines for resubmitting your figure files are available below the reviewer comments at the end of this letter.

If applicable, we recommend that you deposit your laboratory protocols in protocols.io to enhance the reproducibility of your results. Protocols.io assigns your protocol its own identifier (DOI) so that it can be cited independently in the future. For instructions see: https://journals.plos.org/plosone/s/submission-guidelines#loc-laboratory-protocols. Additionally, PLOS ONE offers an option for publishing peer-reviewed Lab Protocol articles, which describe protocols hosted on protocols.io. Read more information on sharing protocols at https://plos.org/protocols?utm_medium=editorial-email&utm_source=authorletters&utm_campaign=protocols.

As the corresponding author, your ORCID iD is verified in the submission system and will appear in the published article. PLOS supports the use of ORCID, and we encourage all coauthors to register for an ORCID iD and use it as well. Please encourage your coauthors to verify their ORCID iD within the submission system before final acceptance, as unverified ORCID iDs will not appear in the published article. *Only*  the individual author can complete the verification step; PLOS staff *cannot*  verify ORCID iDs on behalf of authors.

We look forward to receiving your revised manuscript.

Kind regards,

Chong Liu

Academic Editor

PLOS One

Journal Requirements:

When submitting your revision, we need you to address these additional requirements.

1. Please ensure that your manuscript meets PLOS ONE's style requirements, including those for file naming. The PLOS ONE style templates can be found at

https://journals.plos.org/plosone/s/file?id=wjVg/PLOSOne_formatting_sample_main_body.pdf and https://journals.plos.org/plosone/s/file?id=ba62/PLOSOne_formatting_sample_title_authors_affiliations.pdf

2. We are unable to open your Supporting Information file [Gender_Classification_supplementary.zip]. Please kindly revise as necessary and re-upload.

3. If the reviewer comments include a recommendation to cite specific previously published works, please review and evaluate these publications to determine whether they are relevant and should be cited. There is no requirement to cite these works unless the editor has indicated otherwise.

Additional Editor Comments :

I have now received all of the reviewers’ comments, and overall they are positive. I have the following suggestions:

1. The manuscript title and main text frequently use “gender,” while many of the references and discussion refer to “sex differences.” In fact, the data variable is a self-reported binary male/female category. I suggest using “self-reported binary gender” consistently throughout the manuscript.

2. Please use references from the past five years.

3. After revising the full manuscript, please read it through carefully to check for any potential errors.

[Note: HTML markup is below. Please do not edit.]

Reviewers' comments:

Reviewer's Responses to Questions

**Comments to the Author**

1. Is the manuscript technically sound, and do the data support the conclusions?

The manuscript must describe a technically sound piece of scientific research with data that supports the conclusions. Experiments must have been conducted rigorously, with appropriate controls, replication, and sample sizes. The conclusions must be drawn appropriately based on the data presented.

Reviewer #1: Partly

Reviewer #2: Partly

**********

2. Has the statistical analysis been performed appropriately and rigorously?

Reviewer #1: N/A

Reviewer #2: Yes

**********

3. Have the authors made all data underlying the findings in their manuscript fully available?

The PLOS Data policy requires authors to make all data underlying the findings described in their manuscript fully available without restriction, with rare exception (please refer to the Data Availability Statement in the manuscript PDF file). The data should be provided as part of the manuscript or its supporting information, or deposited to a public repository. For example, in addition to summary statistics, the data points behind means, medians and variance measures should be available. If there are restrictions on publicly sharing data—e.g. participant privacy or use of data from a third party—those must be specified.

Reviewer #1: Yes

Reviewer #2: Yes

**********

4. Is the manuscript presented in an intelligible fashion and written in standard English?

PLOS ONE does not copyedit accepted manuscripts, so the language in submitted articles must be clear, correct, and unambiguous. Any typographical or grammatical errors should be corrected at revision, so please note any specific errors here.

Reviewer #1: Yes

Reviewer #2: Yes

**********

5. Review Comments to the Author

Please use the space provided to explain your answers to the questions above. You may also include additional comments for the author, including concerns about dual publication, research ethics, or publication ethics. (Please upload your review as an attachment if it exceeds 20,000 characters)

Reviewer #1: The paper has a very interesting basic idea, especially that of using "feature importance" in social research. However, there are some problems:

MULTICOLLINEARITY: To make the analysis more robust, given that personality traits are correlated a priori at the theoretical level and that the analyses here use SHAP and permutation, which are unstable with correlated constructs, it is necessary to directly add a multicollinearity analysis to the paper using the correlation matrix (since both SHAP and permutation importance are sensitive) and possibly add conditional permutation and SHAP as a robustness check.

LDA: While not strictly necessary, it would be more robust to add an analysis of the basic applicability assumptions (covariance and distribution), i.e., adding analyses of equal covariance across groups and the data distribution.

RANKING: Although the use of rankings is justified given the different scales, it would be appropriate to add complementary analyses on normalized continuous values.

ADD A PARAGRAPH ON LIMITATIONS: It is strongly necessary to add a paragraph on limitations or include them in the conclusions (but giving them appropriate space), highlighting in more detail all critical aspects, such as the presence of correlations, the size difference between Big 5 and 16pf, data dated 2014, common self-report issues such as social desirability, distinction between sex and gender (binary classification only), sensitivity of model importances , and no control for age, origin, or other factors.

Reviewer #2: In “Gender Prediction from Personality Traits” Cho, Chen and Wallraven ask how much insight do machine learning models bring to understanding personality trait structure relative to linear statistical geometry. They compare the distributions of classic personality traits (Big 5 and 16 personality factors, 16PF) along the male-female gender divide with the relative feature importance of these traits in machine-learning-based gender prediction models. They find that in large cohorts, feature importance is strongly related with univariate or multivariate trait effects, and conclude that machine learning methods recover this same underlying structure. For acceptance in PLOS One, there are seven criteria that must be met (https://journals.plos.org/plosone/s/reviewer-guidelines), which I can now enumerate. To my knowledge (1) these findings are original, (2) they have not been published elsewhere, (3) the experiments, statistics and analyses are technically sound and sufficiently well described, (4) I address below, (5) the article is presented intelligibly and written in standard English, (6) meets applicable ethical standards and (7) adheres to appropriate data availability standards. However, I am unsatisfied with the way the conclusions are reported (criterion 4). The scope of their findings requires careful delineation and their manuscript would benefit substantially from revisions that clarify this scope.

A foundational premise in machine learning is that in sparse data regimes, where dimensionality outnumbers observed data and unbiased models would otherwise fail, the appropriate choice of an inductive bias can enable successful interpolation of unobserved outcomes. This has led to the application of machine learning methods by researchers hoping to discover hidden structure in their own data, often with little attention to the particular choice of inductive bias. However, Wolpert’s (1996, Neural Computation) famous “No Free Lunch” theorem proves that there is no universally optimal learning algorithm across learning objectives, and thus matching the learning algorithm's inductive bias with the learning objective becomes essential. Cho et al. invite the question of whether particular inductive biases are needed at all, but they spend too little time delimiting the scope of their study. This is fine for sophisticated readers who understand the underlying machine learning theory, but places non-specialist readers at risk of seriously misunderstanding the findings.

This study focuses on a particular kind of data in a particular inductive regime where data exceeds dimensionality by four orders of magnitude. Further, the personality trait data in question is by design intended to provide an additive linear factorization of personality variance, which induces a particular data structure. Its geometry is linear in all parameters with topology devoid of intrinsic “holes”, i.e. all combinations of parameters are plausibly observable and there is no configuration that is a priori precluded. In this regime bias-variance tradeoffs are largely moot due to plentiful data, and nonlinear methods offer little advantage. Indeed, the performance Cho et al report for their linear models are nearly indistinguishable from their nonlinear models (Table 2, e.g. logistic regression of gender Big5 yields AUC=0.678 vs. random forests which yield 0.679; note that AUC is the area under the receiver operator characteristic curve and can be interpreted as classification accuracy in a binary forced choice test). Consequently, their conclusion that feature importance in machine learning models is highly correlated with linear statistical metrics like Cohen’s d and Mahalanobis distance is essentially anticipated by the data regime, feature space, and learning objective.

The authors admit in their discussion that the “topology of inter-trait correlations” is a critical factor underlying their findings but spend no time describing what they mean by this. Further, in their introduction they motivate their interest in machine learning based predictions from personality measures by invoking “digital footprints and behavioral traces”, a data regime with differing dimensionality and topology than purpose made research instruments like Big5 and PF16 traits. This leaves non-specialist readers at risk of misunderstanding the scope of their findings and mistakenly generalizing from findings based on a densely sampled linear discrimination boundary to sparsely sampled nonlinear boundaries. The authors’ discussion should deliberately engage with this problem and scope the limits of their findings at least with respect to the kinds of applications they cite for motivation in their introduction.

The necessary changes can be reasonably expected in a revision of this article. In addition, some more minor issues might also be addressed. These are as follows,

1. The abstract would benefit from clarifying that both linear and nonlinear machine learning models were trained. This can be as simple as changing “We trained multiple machine learning models” to “We trained multiple linear and nonlinear machine learning models”.

2. At line 225, recontextualize the resampling for the reader. Instead of writing “Resampling effects were negligible in the 16PF...” Write something like "Choice of resampling method to correct for class imbalance had a negligible effect on model accuracies in the 16PF…". This will help the reader remember what resampling was for after having last read about it several paragraphs prior in the methods section.

3. Lines 239-241 and 249-250 appear to contradict themselves. In the former it sounds like feature ranks are averaged across models and then correlated with statistical dispersion measures (Cohen’s d, Mahalanobis distance, etc.), while in the latter it sounds like feature ranks are correlated with statistical dispersion measures independently for each model class and then correlations are averaged. Based on the methods write up, I believe the authors did the latter. The language should be homogenized to avoid confusion.

4. The claim on lines 316-318 that “the empirical result confirms that this relationship extends … to nonlinear classifiers” is too strong. The empirical results only confirm that this relationship extends to nonlinear classifiers when applied to data with linear discriminant boundaries.

With some revisions to the way the manuscript is written it could be suitable for publication.

**********

6. PLOS authors have the option to publish the peer review history of their article (what does this mean?). If published, this will include your full peer review and any attached files.

If you choose “no”, your identity will remain anonymous but your review may still be made public.

**Do you want your identity to be public for this peer review?**  For information about this choice, including consent withdrawal, please see our Privacy Policy.

Reviewer #1: No

Reviewer #2: **Yes:** Bogdan Petre

**********

[NOTE: If reviewer comments were submitted as an attachment file, they will be attached to this email and accessible via the submission site. Please log into your account, locate the manuscript record, and check for the action link "View Attachments". If this link does not appear, there are no attachment files.]

To ensure your figures meet our technical requirements, please review our figure guidelines: https://journals.plos.org/plosone/s/figures

You may also use PLOS’s free figure tool, NAAS, to help you prepare publication quality figures: https://journals.plos.org/plosone/s/figures#loc-tools-for-figure-preparation.

NAAS will assess whether your figures meet our technical requirements by comparing each figure against our figure specifications.

# Author comment: Author response to Decision Letter 1

**DOI:** 10.1371/journal.pone.0355193.r002

1 Jul 2026

Please see the attached file that contains our detailed point-by-point response.

Attachment Submitted filename: Response to Reviewers.pdf

# Peer review: Decision Letter 1

**DOI:** 10.1371/journal.pone.0355193.r003

**Authors:** Chong Liu

19 Jul 2026

Self-reported binary gender prediction from personality traits: Alignment between machine learning importance and classical effect sizes

PONE-D-26-12774R1

Dear Dr. Wallraven,

We’re pleased to inform you that your manuscript has been judged scientifically suitable for publication and will be formally accepted for publication once it meets all outstanding technical requirements.

Within one week, you’ll receive an e-mail detailing the required amendments. When these have been addressed, you’ll receive a formal acceptance letter and your manuscript will be scheduled for publication.

An invoice will be generated when your article is formally accepted. Please note, if your institution has a publishing partnership with PLOS and your article meets the relevant criteria, all or part of your publication costs will be covered. Please make sure your user information is up-to-date by logging into Editorial Manager at Editorial Manager® and clicking the ‘Update My Information' link at the top of the page. For questions related to billing, please contact billing support.

If your institution or institutions have a press office, please notify them about your upcoming paper to help maximize its impact. If they’ll be preparing press materials, please inform our press team as soon as possible -- no later than 48 hours after receiving the formal acceptance. Your manuscript will remain under strict press embargo until 2 pm Eastern Time on the date of publication. For more information, please contact onepress@plos.org.

Kind regards,

Chong Liu

Academic Editor

PLOS One

Additional Editor Comments (optional):

Reviewers' comments:

Reviewer's Responses to Questions

**Comments to the Author**

1. If the authors have adequately addressed your comments raised in a previous round of review and you feel that this manuscript is now acceptable for publication, you may indicate that here to bypass the “Comments to the Author” section, enter your conflict of interest statement in the “Confidential to Editor” section, and submit your "Accept" recommendation.

Reviewer #2: All comments have been addressed

**********

2. Is the manuscript technically sound, and do the data support the conclusions?

The manuscript must describe a technically sound piece of scientific research with data that supports the conclusions. Experiments must have been conducted rigorously, with appropriate controls, replication, and sample sizes. The conclusions must be drawn appropriately based on the data presented.

Reviewer #2: Yes

**********

3. Has the statistical analysis been performed appropriately and rigorously?

Reviewer #2: Yes

**********

4. Have the authors made all data underlying the findings in their manuscript fully available?

The PLOS Data policy requires authors to make all data underlying the findings described in their manuscript fully available without restriction, with rare exception (please refer to the Data Availability Statement in the manuscript PDF file). The data should be provided as part of the manuscript or its supporting information, or deposited to a public repository. For example, in addition to summary statistics, the data points behind means, medians and variance measures should be available. If there are restrictions on publicly sharing data—e.g. participant privacy or use of data from a third party—those must be specified.

Reviewer #2: Yes

**********

5. Is the manuscript presented in an intelligible fashion and written in standard English?

PLOS ONE does not copyedit accepted manuscripts, so the language in submitted articles must be clear, correct, and unambiguous. Any typographical or grammatical errors should be corrected at revision, so please note any specific errors here.

Reviewer #2: Yes

**********

6. Review Comments to the Author

Please use the space provided to explain your answers to the questions above. You may also include additional comments for the author, including concerns about dual publication, research ethics, or publication ethics. (Please upload your review as an attachment if it exceeds 20,000 characters)

Reviewer #2: In my previous review of this manuscript I was primarily concerned by the omission of any discussion of the inductive regime in which the authors were operating, for example the fact that they had many more observations than parameters, and were working with a linearly factorized feature set. The additions made to the Discussion section of the manuscript have addressed this major concern to my satisfaction. Minor concerns, such as they were, have also been also addressed.

**********

7. PLOS authors have the option to publish the peer review history of their article (what does this mean?). If published, this will include your full peer review and any attached files.

If you choose “no”, your identity will remain anonymous but your review may still be made public.

**Do you want your identity to be public for this peer review?**  For information about this choice, including consent withdrawal, please see our Privacy Policy.

Reviewer #2: **Yes:** Bogdan Petre

**********

# Editor report: Acceptance letter

**DOI:** 10.1371/journal.pone.0355193.r004

**Authors:** Chong Liu

PONE-D-26-12774R1

PLOS One

Dear Dr. Wallraven,

I'm pleased to inform you that your manuscript has been deemed suitable for publication in PLOS One. Congratulations! Your manuscript is now being handed over to our production team.

At this stage, our production department will prepare your paper for publication. This includes ensuring the following:

* All references, tables, and figures are properly cited

* All relevant supporting information is included in the manuscript submission,

* There are no issues that prevent the paper from being properly typeset

You will receive further instructions from the production team, including instructions on how to review your proof when it is ready. Please keep in mind that we are working through a large volume of accepted articles, so please give us a few days to review your paper and let you know the next and final steps.

Lastly, if your institution or institutions have a press office, please let them know about your upcoming paper now to help maximize its impact. If they'll be preparing press materials, please inform our press team within the next 48 hours. Your manuscript will remain under strict press embargo until 2 pm Eastern Time on the date of publication. For more information, please contact onepress@plos.org.

You will receive an invoice from PLOS for your publication fee after your manuscript has reached the completed accept phase. If you receive an email requesting payment before acceptance or for any other service, this may be a phishing scheme. Learn how to identify phishing emails and protect your accounts at https://explore.plos.org/phishing.

If we can help with anything else, please email us at customercare@plos.org.

Thank you for submitting your work to PLOS ONE and supporting open access.

Kind regards,

PLOS ONE Editorial Office Staff

on behalf of

Dr. Chong Liu

Academic Editor

PLOS One
